# 10 years of *Cell Death & Disease*

**DOI:** 10.1038/s41419-020-03287-y

**Published:** 2020-12-12

**Authors:** Mauro Piacentini, Yufang Shi, Hans-Uwe Simon

**Affiliations:** 1 Department of Biology, University of Rome “Tor Vergata”, Rome, Italy; 2 National Institute for infectious Disease IRCCS”Lazzaro Spallanzani”, Rome, Italy; 3 CAS Key Laboratory of Tissue Microenvironment and Tumor, Shanghai Institute of Nutrition and Health, Chinese Academy of Sciences, Shanghai, China; 4 The First Affiliated Hospital of Soochow University and State Key Laboratory of Radiation Medicine and Protection, Institutes for Translational Medicine, Soochow University Medical College, Suzhou, China; 5 Institute of Pharmacology, University of Bern, Bern, Switzerland; 6 Department of Clinical Immunology and Allergology, Sechenov University, Moscow, Russia

*Cell Death & Disease* is celebrating its 10th anniversary. In the last 10 years from its foundation the content of *Cell Death & Disease* has covered an enormous breadth of subjects at the forefront of experimental medicine and clinical practice. The Journal’s mission aims to encompass the breadth of translational implications of cell death in the pathogenesis of major human diseases. To mark this important milestone, we requested updates of the research of those scientists who have significantly contributed in this decade to the activity of the Journal with highly cited publications. These high influential original papers together with topical reviews have led *Cell Death & Disease* to become a successful landmark in science publishing as highlighted by its growing altmetrics and high impact factor (2019 IF 6.3). The central purpose of *Cell Death & Disease* is the publication of original peer-reviewed work that constitutes the true basis for advancing biomedical science. This commemorating review set includes four reviews dealing with hot aspects of cancer such as the role cell death regulators, exosomes, metabolism, and long non-coding RNAs and their possible implications in therapy^1–4^. The fifth contribution encompasses the recent developments in the field of metabolic diseases such as non-alcoholic fatty liver disease (NAFLD), the most common cause of chronic liver disease worldwide^5^. One of the major translational achievement of the cell death field is the development of BCL-2 family protein inhibitors such as Venetoclax (ABT-199, GDC-0199), the first clinically approved drug of this class, which is currently used in the treatment of chronic lymphocytic leukemia (CLL) as well as acute myeloid leukemia (AML)^6^. However, despite remarkable clinical results, a prolonged treatment with the Venetoclax monotherapy leads to drug resistance. The review by Kapoor et al.^1^ analyzes in detail the mechanism of action of BCL-2 inhibition focusing on the acquired resistance to venetoclax as well as on the possibility that tumors initially resistant to Venetoclax become responsive to it following prior therapies. In the last few years, we have also witnessed another revolution in cancer treatment through targeting the tumor microenvironment to enhance the antitumor immunity. The cytotoxic T-lymphocyte-associated antigen 4 (CTLA-4) and programmed death 1 (PD-1) immune checkpoints are negative regulators of T-cell immune function and their inhibition resulting in increased activation of the immune system is the new frontiers in cancer treatment^6^. However, many cancer patients do not respond initially or develop secondary resistance. In their review, Cerezo and Rocchi^2^ discuss how targeting metabolism could help modulate antitumor immunity and exploring the possibility that the metabolic reprogramming in cancer cells may represent a way to favor the antitumor response^7^. Another key factor in the development of cancer tumor resistance to therapy is discussed in the review by Dong et al.^3^ highlighting the contribution of exosomes to drug resistance in breast cancer. The authors dissect out the role of exosomes biogenesis, the influence of their cargos, and the pattern of release in response to drug treatment. In particular, they discuss how proteins or non-coding RNAs contained in exosomes released in the tumor microenvironment by the tumor itself and/or the stromal cells are able to influence drug resistance by altering the metabolism, pro-survival signaling, epithelial-mesenchymal transition, stem-like property of breast cancer. Breast cancer metastasis is the major cause of mortality in female patients^8^. An increasing number of studies published in *Cell Death & Disease* focus on the involvement of long non-coding RNAs (lncRNAs) in cancer metastasis; however, a defined role of these lncRNAs are yet to be clarified^9–11^. The review by Liu et al.^4^ focuses on the lncRNAs functions in breast cancer invasion and metastasis, with particular emphasis on their dual functions for metastasis, their functional mechanisms, their regulatory factors, and the therapeutic promises. The last review by Rada et al.^5^ of this collection does not appear to be related to the previous ones at a first glance. Indeed it discusses a metabolic aspect that is assuming more and more importance nowadays. In fact, non-alcoholic fatty liver disease (NAFLD) is the commonest cause of chronic liver disease world-wide and can progress to cirrhosis and hepatocellular carcinoma^12^. The global prevalence of NAFLD is thought to be constantly increasing, being currently estimated to about 25% of the population^13^. The review by Rada et al. discuss some of the molecular mechanisms that can be responsible for lipotoxicity in NAFLD, including ER and oxidative stress, autophagy, lipoapoptosis, and inflammation. In particular, the review highlights the role of CD36/FAT fatty acid translocase in NAFLD pathogenesis. Clinical studies have shown that CD36 increased levels in the liver of NAFLD patients as well as circulating levels of a soluble form of CD36 (sCD36) that are positively correlated with the histological grade of hepatic steatosis. The authors highlight how CD36 or some of its functional regulators may be a promising therapeutic approach for the prevention and treatment of NAFLD and consequently of cirrhosis and hepatocellular carcinoma. We hope that the readers enjoy this review set which represents just the tip of the iceberg of our past, present, and future commitment to advance translational science. The *Cell Death & Disease* foremost goal is to publish a peer-reviewed scientific journal of the highest quality focused on understanding the mechanistic bases of disease. If we achieve this goal, the merit goes to the authors who have contributed their original work to our Journal and to the Editors and Reviewers who provided the expertise for processing the thousand manuscripts we annually receive. We finally would like to thank all the past and present members of our Editorial Office as well as the hard-working production Editors at Springer/Nature, as they finalize the publication of the high-quality papers to which we became accustomed.
